# Eye movement desensitization and reprocessing (EMDR) therapy or supportive counseling prior to exposure therapy in patients with panic disorder: study protocol for a multicenter randomized controlled trial (IMPROVE)

**DOI:** 10.1186/s12888-022-04320-4

**Published:** 2023-03-14

**Authors:** Bart Endhoven, Klara De Cort, Suzy J. M. A. Matthijssen, Ad de Jongh, Agnes van Minnen, Puck Duits, Koen R. J. Schruers, Eva A. M. van Dis, Angelos M. Krypotos, Lotte Gerritsen, Iris M. Engelhard

**Affiliations:** 1 Department of Clinical Psychology, Utrecht University, PO Box 80140, 3508 TC Utrecht, The Netherlands; 2 Altrecht Academic Anxiety Center, Nieuwe, Houtenseweg 12, 3524 SH Utrecht, The Netherlands; 3 Academic Anxiety Center, Mondriaan/PsyQ, Oranjeplein 10, 6624 KD Maastricht, The Netherlands; 4 Department of Psychiatry and Neuropsychology, Maastricht University, PO Box 616, 6200MD Maastricht, The Netherlands; 5 PSYTREC, Professor Bronkhorstlaan 2, 3723 MB Bilthoven, The Netherlands; 6 Academic Centre for Dentistry Amsterdam, University of Amsterdam and VU University Amsterdam), Gustav Mahlerlaan 3004, 1081 LA Amsterdam, The Netherlands; 7 Behavioural Science Institute, Radboud University, PO Box 9104, 6500HE Nijmegen, The Netherlands; 8 Research Group Health Psychology, PO Box 3726, 3000 Leuven, KU Belgium

**Keywords:** Exposure Therapy, Eye Movement Desensitization and Reprocessing (EMDR) therapy, Supportive Counseling, Randomized Controlled Trial, Anxiety Disorder, Panic Disorder, Cognitive-Behavioral Therapy (CBT)

## Abstract

**Background:**

Exposure-based therapy is the treatment of choice for anxiety disorders, but many patients do not benefit sufficiently from it. Distressing images of threat related to the future or past may maintain the anxiety symptomatology or impede exposure therapy. An intervention that targets threat-related imagery is eye movement desensitization and reprocessing (EMDR) therapy. The main goal of this multicenter randomized controlled trial is to investigate whether EMDR therapy plus exposure therapy, relative to supportive counseling plus exposure therapy, improves treatment efficacy, tolerability, and adherence in patients with panic disorder. In addition, we will examine potential predictors of optimal treatment allocation, mechanisms of change as well as the long term effects of treatment. Finally, we will assess cost-effectiveness.

**Methods:**

A multicenter randomized controlled trial mixed design will be conducted. Participants will be 50 patients, aged ≥ 18, diagnosed with a panic disorder. They will be randomly assigned to one of two conditions: EMDR therapy (i.e., flashforward strategy) or supportive counseling (each consisting of four weekly sessions of 90 min each) prior to exposure therapy (consisting of eight weekly sessions of 90 min each). Assessments will be made pre-treatment (T1), between-treatments (T2), post-treatment (T3), one month post-treatment (FU1) and six months post-treatment (FU2) by an assessor blind to treatment condition. The primary outcome measure is severity of panic-related symptoms. Secondary outcome measures are: tolerability of exposure therapy (initial avoidance, willingness to start exposure therapy, considered drop-out; no-show and drop-out), related symptomatology (generalized anxiety, depression), and functional impairment.

**Discussion:**

The primary goals of this research are to compare the efficacy, tolerability, and adherence of EMDR therapy plus exposure therapy and supportive counseling plus exposure therapy and to identify predictors, moderators, and mediators for treatment success. This multi-center research aims to make a significant contribution to our understanding as to how treatment for patients with anxiety disorders can be optimized, and elucidate who can benefit most from this novel approach.

**Trial registration:**

ISRCTN—ISRCTN29668369: Improving anxiety treatment by modifying emotional memories before real-life exposure. Registered 27 June 2022—retrospectively registered. ISRCTN—ISRCTN29668369.

## Background

Anxiety disorders are among the most common mental disorders, with about one out of 14 people meeting diagnostic criteria at any point of time [1]. These disorders comprise panic disorder, agoraphobia, social anxiety disorder, specific phobia, and generalized anxiety disorder. Their key elements are excessive fear in anticipation or the presence of the feared (innocuous) stimulus/stimuli and avoidance of disorder-related situations [2], which cause personal suffering and/or functional impairment [3]. Annual societal costs associated with anxiety-related disorders are estimated at about €74 billion in the European Union [4] and $42 billion in the United States [5].

Cognitive-behavioral therapy (CBT) is the psychological treatment of choice for anxiety disorders recommended in clinical guidelines [e.g., [6]], and it typically includes a combination of cognitive restructuring and behavioral (e.g., exposure) interventions [6, 7]. During exposure therapy, patients are systematically confronted with stimuli and situations associated with their anxiety disorder (e.g., bodily sensations in case of panic disorder), so that the patient learns that the anticipated feared outcome does not occur (e.g., having a heart attack) [8]. Meta-analyses have shown large effect sizes for CBT in anxiety disorders, especially when exposure strategies are used [9, 10].

A recent meta-analysis, however, shows that exposure-based CBT is moderately efficacious for anxiety disorders compared to other interventions when controlling for non-specific treatment or placebo (pill or psychological) effects [9]. There is substantial room for improvement for at least four reasons. First, about 25–30% of patients refuse to start exposure therapy, because they find it too aversive [11, 12]. Second, about 10–20% of patients drop out prematurely [12–14]. Third, although meta-analyses show substantial effect sizes for (exposure-based) CBT, the response rate (i.e., the percentage of patients showing significant clinical improvement) is around 50% [15]. Finally, little research has focused on relapse after (exposure-based) CBT [16–18], but recent findings suggest that it occurs in about 14% of cases [17]. Additionally, laboratory research has shown that even when exposure-based training is initially successful, learned fear can later return [19]. Taken together, these results underline the need for treatment optimization.

According to prevailing associative learning theories [20], the intensity of learned fear is determined by two factors: the strength of threat expectancy (e.g., expecting that dizziness or heart palpitations are harbingers of having a heart attack) and the mental representation of threat intensity (e.g., having a heart attack). Presumably, exposure therapy focuses specifically on the first factor: maximizing violations of threat expectancy [8]. It is not directly aimed at the second factor: the threat intensity representation [21] such as vivid and upsetting mental imagery of having a heart attack in a supermarket. Such imagery shares similarities with traumatic memories in posttraumatic stress disorder (PTSD) [21, 22]. Indeed, previous research showed that anxiety patients often report vivid and distressing mental images of threat [23, 24], related to past aversive events (“flash-backs”) or, likely more importantly, anticipated future threat (i.e., “flash-forwards”) [25, 26].

If patients with anxiety disorders experience intense threat imagery, they might be too anxious [27, 28] or unwilling to confront feared stimuli [28–30], which could explain the low attrition rates in exposure therapy. Interestingly, laboratory fear conditioning studies have shown that an imagery intervention to modulate threat imagery enhances extinction learning and reduces return of fear [31–33]. Preliminary research has also shown promising results of clinical interventions that specifically focus on threat imagery in anxiety disorders, such as imagery rescripting [34–37] or EMDR therapy [38–40].

EMDR therapy was developed to alleviate the distress associated with traumatic memories [41]. During EMDR therapy, patients recall a traumatic memory while simultaneously focusing on another task (such as making eye movements by tracking the therapist’s fingers). A large body of experimental research suggests that the emotional intensity and vividness of aversive memories can be reduced when the person performs a distracting task that taxes working memory while recalling the aversive memory [42–44]. Because similar neural mechanisms are involved in past and future memories [45], EMDR therapy may also be effective to modulate fear-related future memories. Pre-clinical laboratory research has indeed shown that an EMDR lab analog modulates future-oriented threat memories [26, 46, 47]. Accordingly, a clinical EMDR “flash-forward” protocol has been developed [48].

Several randomized controlled trials found that EMDR therapy focused on past aversive events reduces anxiety symptoms [39, 40], but the majority have methodological limitations, including the risk of bias, small samples, and the lack of an active control condition [38, 40]. No research so far has tested whether modulation of flashforwards reduces anxiety symptoms in itself and/or enhances efficacy, tolerability, and adherence of exposure-based therapy in patients with anxiety disorder. The primary aim of the present study is to test the efficacy, tolerability, and adherence of this promising approach [23, 49]. To control for nonspecific factors, we will use an active, nonspecific and nondirective intervention aimed at offering support (supportive counseling) [50]. Patients with panic disorder will be the focus of the study, because mental images of threat are prominent in this disorder [21]. Furthermore, panic disorder is prevalent, which enhances the study’s feasibility [51].

## Goals of the current study:

The main goal of this multicenter Randomized Controlled Trial is to investigate whether EMDR therapy with exposure therapy, relative to supportive counseling with exposure therapy, improves treatment efficacy, tolerability, and adherence in patients with panic disorder. The primary outcome measures are panic-related symptoms. Secondary outcome measures are: tolerability of exposure therapy (initial avoidance, willingness to start exposure therapy, considered drop-out; no-show and drop-out), related symptomatology (generalized anxiety, depression) and functional impairment. We hypothesize that: (1) EMDR therapy will be more effective in reducing panic-related symptoms, compared to supportive counselling from baseline (T1) to between-treatments (T2). (2) EMDR therapy + exposure therapy will be more effective in reducing panic-related symptoms, compared to supportive counseling + exposure therapy at post-treatment (T3). (3) EMDR therapy will result in higher tolerability of exposure therapy (less initial avoidance, more willingness to start exposure therapy, less considered drop-out; less no-show and actual drop-out) compared to supportive counseling. We further hypothesize that: (4) EMDR therapy will be more effective in reducing related symptomatology (generalized anxiety, depression) and functional impairment, compared to supportive counseling from baseline (T1) to between-treatments (T2). And (5) EMDR therapy + exposure therapy will be more effective in reducing related symptomatology (generalized anxiety, depression) and personal suffering/functional impairment, compared to supportive counseling + exposure therapy at post-treatment (T3).

Hypotheses concerning the following secondary goals will be preregistered on the Open Science Framework [52]. The first goal is to test whether EMDR therapy + exposure therapy results in less return of fear (panic related symptoms, generalized anxiety), compared to supportive counseling + exposure therapy from posttreatment (T3) to follow up (FU1 and FU2). The second goal is to unravel predictors of optimal treatment allocation (EMDR therapy + exposure therapy > supportive counseling + exposure therapy). These include theory-driven variables (reduced extinction learning, less experienced life events, low intolerance of uncertainty, anxiety sensitivity, low worrying, enhanced imagery ability), patient variables (e.g., greater treatment credibility, better working alliance) and therapist factors (e.g., enhanced trait anxiety, better working alliance, greater treatment expectancy). The third goal is to elucidate mechanisms of change (most notably mental threat imagery, encapsulated threat beliefs) of this novel approach (EMDR therapy + exposure therapy). Finally, we will assess cost-effectiveness of the new approach (EMDR therapy + exposure therapy).

## Methods/design

### Study design and general procedure

**Fig. 1:**
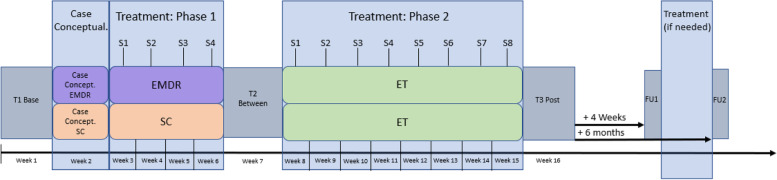
Study design; T1-3: measurements, S1-4/8: sessions, FU1-2: follow up measurements. EMDR: EMDR therapy, SC: supportive counselling, ET: exposure therapy

**Fig. 2:**
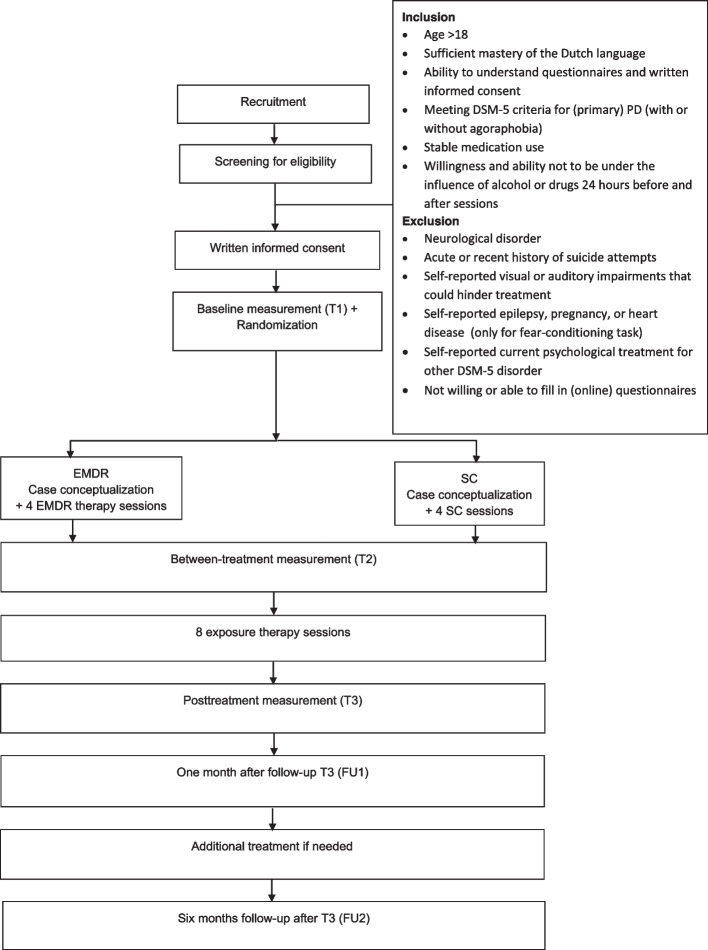
Design flowchart; T1-3: measurements, FU1-2: follow up measurements. EMDR: EMDR therapy, SC: supportive counselling

A multicenter, single blind, randomized controlled trial with a two-arm mixed factorial design is used with two groups (EMDR therapy + exposure therapy, supportive counseling + exposure therapy) and three time points (T1: baseline; T2: between-treatments and; T3: posttreatment). The design is presented in Fig. 1 and a flowchart is presented in Fig. 2.

After baseline measurements (T1), participants will be randomly allocated to one of two conditions: (1) four weekly EMDR therapy sessions followed by eight exposure therapy sessions or (2) four weekly supportive counseling sessions followed by eight exposure therapy sessions. Total study duration is 16 weeks (week 1: baseline measurement T1, week 2: case conceptualization, week 3–6: treatment phase 1; EMDR therapy or supportive counseling, week 7: between-treatments measurement T2, week 8–15: treatment phase 2; exposure therapy, week 16: post-treatment measurement T3). Follow up 1 measurement (FU1) and follow up 2 measurement (FU2) will be conducted one month and six months post-treatment measurement (T3). At FU1, the clinician and participant will determine whether additional treatment is needed. Measurement are conducted by a research assistant blind to treatment allocation. The research protocol has been approved by the Medical Ethical Committee of University Medical Center Utrecht (NL NL73918.041).

### Recruitment

Participants will be recruited at two specialized treatment centers in the Netherlands: Mondriaan/PsyQ Academic Anxiety Center and Altrecht Academic Anxiety center. Clinicians refer patients who potentially meet inclusion criteria after a standard intake procedure. Potential participants are invited for an information session with the research assistant, who examines inclusion and exclusion criteria. After providing informed consent, baseline assessments (T1) will be made. Afterwards, participants are randomized to one of conditions (EMDR therapy + exposure therapy or supportive counseling + exposure therapy). Inclusion will continue until 50 panic disorder patients completed posttreatment (T3).

### Participants

Patients will be eligible to participate in the study if they meet the following criteria: (1) at least 18 years of age; (2) sufficient mastery of Dutch language and able to complete questionnaires and understand informed consent information; (3) meet DSM-5 criteria for (primary) panic disorder (with or without agoraphobia); (4) stable medication for at least six weeks and willingness to keep medication stable during the study period (until FU1); and (5) self-reported ability to refrain from alcohol or drugs 24 h before and after each session. General use of alcohol or drugs will be discouraged. Use of sedating medication (e.g., benzodiazepines) is not a contra-indication, but participants are discouraged to use sedating medication before or after treatment sessions and subsequent days. Use of sedating medication will be registered. Patients will not be eligible if they meet any of the following exclusion criteria: (1) self-reported neurological disorder; (2) acute or recent history of suicide attempts according to the Mini-International Neuropsychiatric Interview (M.I.N.I.) section C; (3) self-reported visual or auditory impairments that could hinder treatment; (4) self-reported epilepsy, pregnancy, or heart disease (only for fear-conditioning task); (5) self-reported current psychological treatment for other DSM-5 disorder; and (6) not willing or able to fill in (online) questionnaires.

### Randomization

After baseline measures (T1) participants are randomized to one of the two conditions by a research assistant who does not conduct assessments, using computerized randomization (REDCAP) [53, 54]. Stratified randomization will be used to reduce bias by treatment center [55], to guarantee a balanced distribution of participants across conditions.

### Sample size

For the primary objectives of efficacy, tolerability, and adherence, the sample size was calculated using G*power 3.1 [56] using a 2 (groups) × 2 (time) repeated-measures ANOVA. Given the active control condition, we expect a medium effect size [9]. For a between group effect size of f = 0.25, alpha 0.05, power 0.80, 0.5 correlation among repeated measures and 1.0 non-sphericity correction, a total sample size of 98 participants is needed for two-sided testing. Although no formal method is available to determine a repeated-measures ANOVA sample size using Bayesian statistics, approximations for t-test are available [57]. For a one-sided *t*-test with a power of 0.80 and a Bayes factor of 3 (indicating evidence for the data coming from the alternative than the null hypothesis), 87 participants are needed and for a Bayes factor of 5, 99 participants are needed. Originally, we aimed to recruit 100 participants (see pre-registration: ISRCTN - ISRCTN29668369). However, the COVID-19 pandemic has seriously impacted the number of referrals of patients with panic disorder. To increase the feasibility of the study, we formulated directional hypotheses and decided to use one-sided testing, which reduces our sample size to 50 participants [58]. This change was approved by the ethical committee on February 8 2023. Participants who will not show up to therapy sessions in the first two weeks of treatment will be considered non-compliant and will be described as dropouts. Dropouts will not be excluded from intent to treat analyses, but will be replaced by additional participants to preserve statistical power. The completer sample size will be sufficient to address secondary objectives.

### Interventions

Two standardized treatments with an equal duration will be used before (standardized) exposure therapy: EMDR therapy and supportive counseling. Sessions will be provided face-to-face or if that is not possible by video conferencing.

#### EMDR therapy

This study uses the Dutch EMDR 2021 protocol (Dutch translation of Shapiro’s standard EMDR protocol) [41, 59]. Our case conceptualization strategy will be based upon the central question as to what flashforward drives the patient’s panic disorder. First, the patient’s flashforward will be assessed (i.e., the mental picture that displays the most terrible outcome of a panic attack) and if this is not available to the patient, the patients is helped to create such a flashforward. Second, memories that may have contributed to the onset or persistence of the disorder will be ordered hierarchically based on subjective units of disturbance (SUD) scores. For the processing of patients’ flashforwards with EMDR therapy, the Flashforward procedure will be used [48]. During EMDR sessions, therapists aim to tax working memory maximally. That is, rather than just making eye movements, the therapist induces fast eye movements, tapping, calculations, backward spelling etc. [42–44], an approach that is termed EMDR 2.0 [60]. Multiple targets might be desensitized per session depending on the velocity of SUD decrease. If online treatment is required, a secure online EMDR therapy application (Digital_EMDRapp) will be used as a working memory task [61]. In the app, different reaction time tasks with visual (ball) and auditory (sound) stimuli can be manipulated (pattern, color and speed) and administered by the clinician. This program will be made available free of charge for this project by the company MOOVD.

#### Supportive counseling

Supportive counseling is a non-directive psychotherapeutic procedure that focuses on discussing topics that are relevant to the patient in daily life (e.g., family, daily activities, work). The clinician offers support by attentive listening. A protocol was developed based on the protocols used by Bryant and colleagues (1998) [50] and Boelen and colleagues (2007) [62], to match session duration in the EMDR therapy condition and other non-specific treatment effects, such as therapeutic alliance, time and contact with a therapist [63]. In earlier randomized controlled trials, supportive counseling slightly lowered anxiety symptoms in patients [63], so it has been used as a credible active control intervention. During the supportive counseling case-conceptualization session, threat-related imagery will be assessed the same way as in the EMDR therapy condition, so treatment effects can be attributed to the interventions, instead of the case formulation [64]. Next, a supportive counseling case conceptualization will be made, by making an inventory of potential topics the patient wants to discuss. During the sessions, the patient will be free to change the subject to “whatever topic that’s on their mind” [50, 62]. In supportive counseling, clinicians take an emphatical stance and offer support by using the following steps. (1) Ask an open question: “What do you want to discuss right now?”, (2) deepen the conversation by active listening and open questions, (3) paraphrase and reflect during the conversation, (4) summarize the conversation at the end of the conversation, (5) compliment the patient for the hard work, (6) and end the conversation. In order to study working mechanisms, clinicians are explicitly instructed not to focus on specific CBT procedures (i.e., cognitive restructuring, emotional processing of adverse life events, or behavioral exposure) [64].

#### Exposure therapy

During exposure therapy, the patient is systematically and repeatedly confronted with feared but safe stimuli (e.g., interoceptive cues, objects, situations), with the goal that the patient learns that the anticipated feared outcome does not occur (e.g., getting a heart attack) [8]. Exposure therapy will focus on maximizing threat expectancy violation [8]. For panic disorder, the protocol will be based on the Dutch protocol by Kampman, Keijsers and Hendriks (2017) [65]. It involves psycho-education, interoceptive exposure (e.g., with CO_2_ or physical exercises) and exposure in vivo [66]. To maximize expectancy violation, cognitive techniques prior to exposure are not used, as recommended by Craske and colleagues [8].

### Therapists and training

Therapists participating in the trial will be psychologists, psychotherapists or psychiatric nurse specialists (master degree, advanced mental health qualifications) and have all completed at least a CBT basic course (accredited by the Dutch CBT association) and an EMDR basic course (accredited by the Dutch EMDR association). Therapists are employed in specialized anxiety disorders clinics (Altrecht and Mondriaan). For this study, clinicians will receive four hours of training in exposure techniques (focusing om maximizing threat-expectancy violation), four hours of training in EMDR (focusing on case conceptualization in anxiety disorders and maximizing working memory taxation) and four hours of training in supportive counseling (focusing on non-directive support) by experts in the field. To reduce bias, all therapists will be trained and will participate in all three interventions. To reduce expectancy bias, clinicians will be given the following rationale “EMDR therapy and supportive counseling are both new innovative interventions for panic disorder based on the latest research, but with a different focus. Instead of searching for the best therapy on group level, we will use the latest statistical analysis to unravel what works for whom on an individual level using clinical profiles”. Treatment expectancy of therapists will be assessed. To obtain high treatment quality and to check whether the training was sufficient, supervisors will check case-conceptualizations, session reports, and session recordings.

### Supervision and fidelity checks

Case conceptualizations and session reports will be checked after each session and feedback will be provided by experts in the field (EMDR therapy: AdJ, SM; supportive counseling: PD, BE; exposure therapy: AvM, KdC). Supervisors of exposure therapy are blind to the patient’s condition. Clinicians are instructed to report deviations from the protocol. To further improve treatment integrity, online group supervision sessions are provided for all treatments two to four times a year. Treatment sessions will be recorded on audio (supportive counseling and exposure therapy sessions) or video (EMDR therapy sessions and the behavioral avoidance tasks), unless patients do not give permission for this. An independent rater will assess a selection of the video and audiotapes to assess treatment fidelity.

### Early completion

Early completion is defined as: (1) a score of 0 on weekly assessment scores (General Anxiety Disorder (GAD 7) and Panic Disorder Severity Scale (PDSS-SR) (2) in case of EMDR therapy, a SUD of 0 for all targets part of the case conceptualization (3) and no avoidance on the behavioral avoidance task.

### Instruments

#### Goal 1: investigate whether EMDR therapy + exposure therapy vs supportive counseling + exposure therapy improves treatment efficacy, tolerability, and adherence

The primary and secondary measures to examine goal 1 are presented in Table 1.

**Table 1:** Primary and secondary measures to examine goal 1: investigate whether EMDR therapy + exposure therapy vs supportive counseling + exposure therapy improves treatment efficacy, tolerability, and adherence

	**T1**	**Tp1**	**T2**	**Tp2**	**T3**	**FU1**	**FU2**
**Demographics and assessment of eligibility for inclusion**							
Demographics (8)	X	-	-	-	-	-	-
Mini-International Neuropsychiatric Interview Simplified (MINI-S-DSM-5)	X	-	-	-	-	X	-
**Adherence rates**							
Drop out and no-show	-	X	-	X	-	-	-
**Primary outcome: Efficacy panic disorder symptomatology**							
Panic Disorder Severity Scale (PDSS-SR)*(7)	X	X	X	X	X	X	X
Panic Agoraphobia Scale (PAS) (14)	X	-	X	-	X	X	-
Subtle Avoidance Frequency Examination- Agoraphobia (SAFE-A) (40)	X	-	X	-	X	X	X
Fear Questionnaire- Agoraphobia (FQ-A) (5)	X	-	X	-	X	X	X
Bodily Sensation Questionnaire (BSQ) (17)	X	-	X	-	X	X	X
**Secondary outcomes: Tolerability of exposure therapy**							
Exposure willingness (1)	-	X	-	X	-	X	X
Intention to stop treatment (1)	-	-	X	-	X	-	-
Behavioral avoidance task	-	-	-	X	-	-	-
**Secondary outcomes: Related symptomatology and functional impairment**							
General Anxiety Disorder 7 (GAD 7) (7)	X	X	X	X	X	X	X
Patient Health Questionnaire (PHQ-9) (9)	X	X	X	X	X	X	X
Work and Social Adjustment Scale (WSAS) (5)	X	-	X	-	X	X	X
**Secondary outcomes: Treatment satisfaction**							
Client Satisfaction Questionnaire (CSQ-8) (8) and Net Promotor Score (NPS) (1)	-	-	X	-	X	-	-

Note: *(nr.)* number of items, *T1* Baseline, *Tp1* Treatment phase 1 *(EMDR therapy or supportive counseling) T2* Between-treatments, *Tp2* Treatment phase 2 (exposure therapy), *T3* Post-treatments, *FU1* follow-up 1, *FU2* follow-up 2

### Demographics and study eligibility

#### Demographics:

Eight demographic variables will be assessed: age, sex, cultural background, educational level, native language, marital status, familiarity with the treatments and age of panic disorder onset.

#### Mini-International Neuropsychiatric Interview-Simplified for DSM-5 (MINI-S-DSM-5):

To assess panic disorder and other diagnoses, the Dutch translation [67] of the MINI-S- interview [68, 69] version 1.1 (adapted to DSM-5) is administered by an independent psychologist.

### Primary outcome variables: efficacy

#### Panic Disorder Severity Scale- self-report form (PDSS-SR):

The PDSS-SR [70–72] is a 7-item questionnaire to assess the severity of panic disorder. Patients rate the frequency, severity and interference of panic attacks in the last week on a 5-point Likert scale (0 = *no interference*, 5 = *extreme interference*).

#### Panic *Agoraphobia Scale (PAS)*:

The PAS [73] is a 14-item interview to assess panic disorder severity. The severity of panic disorder is rated using a 5-point Likert scale (0 = *not at all*, 4 = *severe*) for each item. As a minor addition the frequency of panic attacks as well as the number of avoided places is registered.

#### Subtle Avoidance Frequency Examination – agoraphobia (SAFE-A):

The SAFE-A is a 40-item questionnaire to assess the frequency of subtle avoidance behavior in agoraphobia [74, 75], using a 5-point Likert scale (0 = *never*, 5 = *always*) and when safety behavior is performed on a 3-point Likert scale (1 = *before the situation*, 2 = *during the situation*, 3 = *during a panic attack*).

#### Fear Questionnaire-*Agoraphobia (FQ-A)*:

The FQ-5 [76, 77] is a 5-item questionnaire to assess avoidance behavior for various places (e.g. busy shop, public transport), using a 9-point Likert scale (0 = *I never avoid this*, 8 = *I always avoid this*).

#### Bodily Sensation Questionnaire (BSQ):

The BSQ [78] is a 17-item questionnaire to assess fear of bodily sensations in panic disorder, using a 5-point Likert scale (1 = *not afraid*, 5 = *extremely afraid*).

### Secondary outcome variables: Tolerability of exposure therapy

#### Willingness to start exposure therapy:

Willingness to start exposure therapy will be assessed with one item developed for this study using a Visual Analogue Scale (VAS) (0 = *not willing to do all exposure exercises,* 100 = *willing to do all exposure exercises*).

#### Intention to stop treatment:

Intention to stop treatment will, retrospectively after treatment, be assessed with one item developed for this study, using a VAS (0 = *not at all,* 100 = *very much*).

#### Behavioral avoidance task:

Behavioral avoidance will be assessed with a standardized task during the first and last exposure therapy session, in which the patient is exposed to self-induced bodily sensations (e.g., self-induced dizziness, accelerated heart rate and hyperventilation or physical exercises) [79]. Clinicians rate the level of avoidance on a standardized scale (developed for this study) based on the number of tasks completed and task difficulty. Afterwards, videotapes will be rated with the same scale by blind assessors.

### Secondary outcome variables: Related symptomatology and functional impairment

#### General Anxiety Disorder 7 (GAD-7):

The GAD-7 [80, 81] is a 7-item questionnaire to measure the frequency of anxiety symptoms on a 4-point Likert scale (0 = *not at all*, 3 = *almost every day*) for the past week.

#### Patient Health Questionnaire (PHQ-9):

The PHQ-9 [81–83] is a 9-item questionnaire measuring the frequency of depression symptoms on a 4-point Likert scale (0 = *not at all*, 3 = *almost every day*) for the past week.

#### Work and Social Adjustment Scale (WSAS):

The WSAS [84, 85] is a 5-item self-report questionnaire to assess disability in daily life (e.g., work, home management, social activities) on a 9-point Likert scale (0 = *no disability*, 8 = *severe disability*).

### Secondary outcome variables: Treatment satisfaction

#### Client Satisfaction Questionnaire (CSQ-8):

The CSQ-8 [86–88] is an 8-item self-report questionnaire to assess treatment satisfaction on a 4-point Likert scale (1 = *totally disagree*, 4 = *totally agree*).

#### Net Promoter Score (NPS):

Patients are asked to give an overall grade for the treatment [89], on an 11-point Likert scale (0 = *very bad treatment*; 10 = *very good treatment*).

#### Instruments goal 2: unravel theory-driven variables (e.g., traits and clinical profiles) and non-specific patient and therapist factors predicting treatment outcome and optimal treatment allocation

Table 2 presents additional measures to examine secondary goals.

**Table 2:** Additional measures to test goal 2: unravel theory-driven variables (e.g., traits and clinical profiles) and non-specific patient and therapist factors predicting treatment outcome and optimal treatment allocation

	**T1**	**Tp1**	**T2**	**Tp2**	**T3**	**FU1**	**FU2**
***Questionnaires for all patients***							
Survey of Autobiographical Memory (SAM) (14)	X	-	-	-	-	-	-
Impact of Event Scale past (15)/future (15)	X	-	X	-	X	X	X
List of Threatening Experiences (LTE) (12)	X	-	-	-	-	-	-
Dutch credibility and expectancy scale (CEQ) (8)		X		X	-	-	-
Working Alliance Inventory-12 (WAI-12) (12)	-		X		X	-	-
Intolerance of Uncertainty Scale (IUS) (12)	X	-	-	-	-	-	-
Anxiety Sensitivity Index (ASI) (18)	X	-	-	-	-	-	-
Penn State Worry Questionnaire (PSWQ) (16)	X	-	-	-	-	-	-
***Computer task***							
De novo fear conditioning task	X	-	-	-	X	-	-
***Questionnaires for all therapists***							
Demographics (10)	X	-	-	-	-	-	-
Attitudes Toward Psychotherapy Treatment Manuals (ATPTM) (19)	X	-	-	-	-	-	-
Evidence-Based Practice Attitude Scale (EBPAS) (15)	X	-	-	-	-	-	-
Credibility Scale (CS) (5)	X	-	-	-	-	-	-
Predicting treatment outcome survey (9)	-	X	-		-	-	-
Working Alliance Inventory-12 (WAI-12) (12)	-	X	-	X	-	-	-
Optimal exposure (2)	-	-	-	-	X	-	-
***Assessment therapists***							
Treatment integrity session reports	-	X	-	X	-	-	-

*Note: (nr.)* number of items, *T1* Baseline*, Tp1* Treatment phase 1 (EMDR therapy or supportive counseling)*, T2* Between-treatments*, Tp2* Treatment phase 2 (exposure therapy)*, T3* Post-treatments*, FU1* follow-up 1*, FU2* follow-up 2

### Patient predictors, mediators and moderators

#### Survey of Autobiographical Memory (SAM)>:

The SAM [90] is a self-report inventory designed to assess naturalistic episodic autobiographical, semantic and spatial memory, as well as prospection or future thinking using a 5-point Likert scale (1 = *strongly disagree*, 5 = *strongly agree*). We include the episodic and future thinking subscales only (14 items).

#### Impact of past event scale (IES):

The IES [91, 92] is a 15-item questionnaire to assess the frequency and avoidance of intrusive mental imagery of past events in the last week, using a 5 point Likert scale (1 = *not at all*, 5 = *extremely*).

#### Impact of future event scale (IFES):

The IFES [25] is a 24-item questionnaire to assess the frequency and avoidance of intrusive mental imagery for future events in the past week, using a 5 point Likert scale (0 = *not at all*, 4 = *very strong*). We administer the intrusion and avoidance subscales (15 items).

#### List of Threatening Events (LTE):

The LTE [93] is a 12-item questionnaire to assess experienced life events, participants indicate if the life event occurred (*yes/no).*

#### Dutch credibility and expectancy questionnaire (CEQ):

The CEQ [94] is a 6-item questionnaire to assess treatment credibility and treatment expectancy. Patients rate treatment credibility and -expectancy on a 9 point Likert scale (1 = *not at all*, 9 = *totally*). Expected levels of improvement are rated on a 0–100 VAS (0 = *no expected improvement*, 100 = *totally recovered*).

#### Working Alliance Inventory (WAI-12):

The WAI-12 [95, 96] is a 12-item questionnaire to assess working alliance, rated on a 5-point Likert Scale (1 = *(almost) never*, 5 = *always*).

#### Intolerance of Uncertainty Scale (IUS):

The IUS [97, 98] is a 12-item questionnaire to assess intolerance of uncertainty, using a 5-point Likert scale (1 = *not at all*, 5 = *totally*).

#### Anxiety Sensitivity Index (ASI):

The ASI [99, 100] is an 18-item questionnaire to assess anxiety sensitivity using a 5-point Likert scale (1 = *(almost) never*, 5 = *always*).

#### Penn State Worry Questionnaire (PSWQ):

The PSWQ [101, 102] is a 16-item questionnaire to assess worrying, using a 5-point Likert scale (1 = *not characteristic at all*, 5 = *very characteristic*).

#### De novo fear conditioning task:

Individual differences in fear extinction will be assessed with a differential fear conditioning computer task, including four phases: 1) habituation; 2) acquisition; using a scream as aversive stimulus [103]; 3) extinction; 4) generalization of extinction. Patients will be asked to rate their distress (phases 1–4) and scream expectancy (phases 2–4) [103–105]. More information on this task can be found in a pre-registration validating this task in a non-clinical sample (https://osf.io/axwyd).

### Clinician: predictors, mediators and moderators

#### Demographics and expertise:

Based on earlier studies [106, 107], we will assess age, gender, years of expertise, main profession, clinical orientation, and memberships of professional associations. Clinicians rate their own perceived skills compared to other clinicians on two 0–100 VASs (0 = *poorest*, 100 = *the best*).

#### Attitudes Toward Psychotherapy Treatment Manuals (ATPTM):

The ATPTM [108] is a 19-item questionnaire to assess clinician's attitudes towards psychotherapy treatment manuals, using a 5-point Likert scale (0 = *strongly disagree*, 4 = *strongly agree*).

#### Evidence-Based Practice Attitude Scale (EBPAS:

The EBPAS [109] is a 15-item questionnaire to assess clinician's attitudes towards evidence-based practice, using a 5-point Likert scale (0 = *strongly disagree*, 4 = *strongly agree*).

#### Credibility Scale (CS):

The CS [110] is a 5-item questionnaire to assess to what extent clinicians perceive the intervention as credible, using a 7-point Likert scale (1 = *not at all*, 7 = *extremely*).

#### Predicting Treatment Outcome Survey (PTOS):

The PTOS is a self-developed 9-item questionnaire to assess to what extent clinicians predict treatment outcome and whether treatment is suitable for a patient, based on a questionnaire developed by Van Minnen and colleagues [106].

#### Working Alliance Inventory (WAI-12):

The WAI-12 [95, 96] is a 12-item questionnaire to assess working alliance, rated on a 5-point Likert Scale (1 = *(almost) never*, 5 = *always*).

#### Optimal exposure:

A 2-item self-developed clinician-administered questionnaire will be used to assess if optimal exposure was hindered using a 7-point Likert scale (1 = *not hindered*, 7 = *severely hindered*) and reasons why optimal exposure could not take place (e.g. *COVID-19 measures, avoidance, illness, too difficult, other*).

#### Instruments goal 3: elucidate mechanisms of change of this novel approach (EMDR therapy + exposure therapy)

### Mental imagery

#### Imagery interview and questionnaire:

The imagery interview is used to assess mental imagery related to panic disorder during the case conceptualization session and the between treatments measurement (T2). It is structured and combines sections of the Waterloo Images and Memories Interview (WIMI) [111], the imagery interview by Boterhoven de Haan and colleagues [112] and the Memory Characteristics Questionnaire [113]. First, patients will be asked to choose which disorder-related memory and flashforward is most distressing. Next, they will be asked to describe the flashforward and memory, in as much detail as possible and rate the images’ qualities on several dimensions, such as vividness, sensory quality, complexity etc. using a 7-point Likert scale. Finally, credibility of the encapsulated belief statement that the mental image depicts is rated using a VAS scale (0 = not at all, 100 = extremely).

#### Instruments goal 4: assess cost-effectiveness of the new approach (EMDR therapy + exposure therapy)

### Quality of life and cost-effectiveness

#### EuroQol (EQ-5D):

The EQ-5D [114] is a widely used 5-item questionnaire to assess quality of life. Impairment on several factors (e.g., mobility, self-care, daily activities) is measured on a 5-point Likert scale (1 = *no impairment*, 5 = *extreme impairment*). Patients also rate their current health on a thermometer ranging from 0 (*worst health imaginable*) to 100 (*best health imaginable*).

#### Additional treatment: Treatment Inventory of Costs (TIC-P):

The TIC-P [115] is an 84-item questionnaire to assess medical consumption (16 items), productivity losses (11 items), and chronic diseases (2).

The EQ-5D will be assessed during baseline (T1), between-treatments (T2), post-treatments (T3) and follow-up measures (FU1, FU2) and the TIC-P will be assessed at baseline (T1) and follow-up measures (FU1, FU2).

### Data analysis

Data of all randomized participants will be analyzed on intent-to-treat basis as well as the completer sample. The primary outcomes (panic disorder symptomology) as well as secondary outcomes (tolerability and adherence) will be analyzed using a mixed regression model, using baseline scores as a covariate. This enables us to identify any fixed or random effects resulting from the treatments and changes over time. To analyze count date (e.g., drop-out, no show), Poisson or negative binomial regression will be used. Data analysis strategies for the secondary goals (goals 2–4) will be pre-registered in prior to analysis in Open Science Framework [52].

## Discussion

We present a study protocol for the multicenter IMPROVE randomized controlled trial, examining whether EMDR therapy + exposure therapy vs. supportive counselling + exposure therapy improves treatment efficacy, tolerability, and adherence in patients with panic disorder. To our knowledge, this is the first study that specifically focusses on desensitizing future as well as past oriented threat-imagery, prior to exposure therapy. Furthermore, this randomized controlled trial will focus on theory-driven as well as non-specific patient and therapist factors predicting treatment outcome and optimal treatment allocation. By focusing on hypothesized underlying mechanisms of change and the long term effects of treatment we will broaden our understanding of treatment processes, potentially paving the way towards the era of personalized treatment. Lastly, cost-effectiveness of the new approach (EMDR therapy + exposure therapy) will be assessed when proven effective.

The study design has several strengths. First, a parsimonious principle was used in the development of the intervention by taking a mechanistic approach [116]. Instead of developing a new treatment, we aim to improve the best available treatment. As suggested by Clark [117], our research started with phenomenological observations [21, 22] followed by years of experimental research in our lab exploring the mechanisms involved [26, 46, 47], so our intervention is strongly grounded in theory as well as experimental research [42–44]. As a next step, we will now test the intervention in practice. Hereby, identifying predictors, mediators and moderators of treatment that could help us develop new theoretical models and aid diagnostic- and treatment decision-making [64, 116], paving the way into the era of personalized medicine [118].

Secondly, we expect to recruit a culturally and socioeconomically diverse sample. No strict exclusion criteria are used and clinics are in large cities across the Netherlands, which enhances the external validity of our results [119, 120]. Given that dropouts are common in this population [11–14], we will use an intention to treat analysis. Reasons for dropout will be monitored as attrition rates are an important outcome measure in this study. Validity of the trial is further improved by using standardized measures and by letting the assessments being conducted by researchers blind to treatment condition. Furthermore, the sample size should provide sufficient power for the primary- and secondary aims to enable meaningful conclusions.

Researcher allegiance bias is problematic in psychotherapy research [121, 122], including studies about EMDR therapy efficacy in anxiety disorders [38, 40]. Therefore, we follow recommendations to reduce research allegiance bias, such as having a collaborative team with mixed allegiances [121]. Study therapists are trained and supervised for the same amount of time for all treatment conditions, and treatment integrity will be checked [123]. To reduce expectancy biases in patients and clinicians a strong rational for all treatments, including supportive counselling, is provided. Furthermore, we use identical procedures (including EMDR-case conceptualization) and treatment duration between conditions. Treatment credibility and expectancy for all treatments are monitored for all patients and study-therapists.

### Limitations

No waiting list control group is used, which could be considered a limitation [124, 125], given that it can serve as a valuable benchmark in pilot studies for a new intervention [123], but it is not necessary in this study for at least four reasons. First, effect sizes for EMDR therapy, supportive counseling and CBT in anxiety disorders have already been established [9, 38, 40], so a benchmark is not needed and could artificially inflate effect sizes [126, 127]. Second, it could also result in unwanted nocebo effects [125, 128, 129]. Third, it can create ethical dilemmas [125, 130] and potentially raise the threshold to participate in a trial resulting in selection bias [127, 131]. Finally, it can reduce study or treatment satisfaction [127, 132] which may influence attrition rates [132].

Furthermore, no direct comparison between EMDR therapy and exposure therapy is made [123]. However, instead of finding a substitute for exposure, we aim to improve it beyond common factors by studying mechanisms of action. So, the question whether EMDR therapy is more effective compared to exposure therapy is beyond the scope of this study.

Although knowledge of the long-term effects of CBT is urgently needed [16, 18] only a six month instead of a one- or two-year follow-up measurement is included. After follow-up 1 (FU1; one-month posttreatment) patients will receive additional treatment if needed (also for co-morbid disorders), as we aim to include a representative sample. Therefore, long-term effects can not solely be attributed to the trial interventions. We could not circumvent this issue due to ethical considerations.

Finally, as this study is an efficacy trial, highly trained and supervised professionals provide the treatment, therefore effectiveness research is still needed after the trial [133]. To promote the dissemination of the results a treatment protocol will be published separately.

To recapitulate, many anxiety patients do not profit sufficiently from CBT, underlining the urgent need for treatment optimization. Threat-related imagery may play a maintaining role in the disorder or impede exposure therapy. Currently, imagery (especially future-oriented) is not targeted in standard CBT for anxiety disorders. Therefore, we aim to assess whether EMDR therapy with exposure therapy, relative to supportive counseling with exposure therapy, improves treatment efficacy, tolerability, and adherence in patients with panic disorder. The results of this study may have important clinical implications for the treatment of patients with anxiety disorders, as well as a better understanding of the mechanisms of action paving the way to personalized medicine.

### Trial status

Currently recruiting participants.

### Protocol version

Version 1: original, issue date 26–08-2020. Version 2: amendment adding treatment integrity (video recordings), issue date 26–05-2021. Version 3: amendment changing information letter and adding time stamps in questionnaire response, issue date 27–07-2021. Version 4: amendment to 1) reduce the sample size for panic disorder from *N* = 100 to *N*= 50 to enhance feasibility of the study and adapting statistical analyses accordingly (one-sides testing instead of two sided testing), 2) terminate a RCT in social anxiety disorder, due to a low influx of participants, 3) the option for participants not to consent with audio and video recordings, 4) change the session frequency (once instead of twice weekly), 5) stopping the Experience Sampling Measurements, 6) enable videoconferencing aside from the pandemic restrictions, 7) report the study progress, issue date 08-02-2023.

## Data Availability

To promote open science, this trial is retrospectively registered ISRCTN—ISRCTN29668369: Improving anxiety treatment by modifying emotional memories before real-life exposure including the (main) hypotheses. Materials (in Dutch) used in the study as well as all the articles that are a product of this trial will be made available after trial completion in the trial registry. The World Health Organization trial registration data set guidelines (SPIRIT guidelines) will be used to register the data set [134, 135]. To promote data quality, a secured data base (REDCAP) will be used [53, 54]. For further details about data storage and security, see the original IRB-proposal. Data sharing for this study is not applicable yet, because no datasets have so far been generated or analyzed.

## Abbreviations

ASI: Anxiety Sensitivity Index
ATPTM: Attitudes Toward Psychotherapy Treatment Manuals
BSQ: Bodily Sensation Questionnaire
CBT: Cognitive behavioral therapy
CEQ: Dutch credibility and expectancy scale
CS: Credibility Scale
CSQ-8: Client Satisfaction Questionnaire
EBPAS: Evidence-Based Practice Attitude Scale
EMDR: Eye movement desensitization and reprocessing
EQ-5D: EuroQol
FQ-A: Fear Questionnaire-Agoraphobia
FU(nr.): Follow up
GAD-7: General Anxiety Disorder 7
IES: Impact of past event scale
IFES: Impact of future event scale
IUS: Intolerance of Uncertainty Scale
LMM: Linear Mixed Models
LTE: List of Threatening Events
MINI-S-DSM-5: Mini-International Neuropsychiatric Interview-Simplified for DSM-5
MLR: Mixed Logistic Regression
NPS: Net Promotor Score
Nr.: Number
PAS: Panic Agoraphobia Scale
PDSS-R: Panic Disorder Severity Scale- self-report form
PHQ-9: Patient Health
PSWQ: Pen State Worry Questionnaire
PTOS: Predicting Treatment Outcome Survey
REDCAP: Research Electronic Data Capture
SAFE-A: Subtle Avoidance Frequency Examination – agoraphobia
SAM: Survey of Autobiographical Memory
SUD: Subjective unit of distress
T(nr.): Assessment
TIC-P: Treatment Inventory of Costs
VAS: Visual Analogue Scales
VOC: Validity of cognition
WAI-12: Working Alliance Inventory
WIMI: Waterloo Images and Memories Interview
WSAS: Work and Social Adjustment Scale
